# Intracapsular tonsillectomy in adults with obstructive sleep apnoea and large tonsils

**DOI:** 10.1007/s00405-026-10497-1

**Published:** 2026-08-03

**Authors:** Henrik M. Sjöblom, Jenny Knubb, Miika Suomela, Lauriina Lustig-Tammi, Jaakko M. Piitulainen

**Affiliations:** 1 Department of Otorhinolaryngology – Head and Neck Surgery, Division of Surgery and Cancer Diseases, Turku University Hospital, Turku, Finland; 2 Department of Clinical Neurophysiology, Turku University Hospital, Turku, Finland; 3 Department of Medicine, University of Turku, Turku, Finland; 4 4Department of Otorhinolaryngology, Satasairaala, Pori, Finland; 5 Department of Otorhinolaryngology, PO Box 52, Turku, 20521 Finland

**Keywords:** tonsillectomy, tonsillotomy, intracapsular, obstructive sleep apnoea, tonsil hypertrophy

## Abstract

**Purpose:**

Conventional (extracapsular) tonsillectomy is a well-established treatment for obstructive sleep apnoea (OSA) in adults with tonsillar hypertrophy. Our objective was to determine whether an intracapsular tonsillectomy (ICTE) with a 90% removal of tonsils could be sufficient. The secondary objective was to evaluate if 10 days of sick leave is enough for adults after ICTE.

**Methods:**

This prospective cohort study included adults aged 16–65 years with moderate to severe OSA (apnoea–hypopnoea index [AHI] ≥ 15) and tonsillar hypertrophy. All patients underwent ICTE using coblation. The main outcome was surgical success as defined by a > 50% decrease in the overall AHI and a decrease in the overall AHI to < 20. Surgical cure was defined by a decrease in the overall AHI to < 5. To track recovery, participants completed the Brief Pain Inventory. Patient reported outcomes were assessed using the Insomnia Severity Index (ISI), Epworth Sleepiness Scale (ESS), and Glasgow Benefit Inventory (GBI).

**Results:**

Twenty patients underwent surgery, and sixteen completed the 6-month follow-up. The surgical success rate was 68.8%, and the surgical cure rate was 43.8%. Mean (± SD) AHI lowered from 37.4 (± 19.8) to 11.8 (± 16.1) events per hour (*p* = .003). Mean AI lowered from 13.3 (± 13.9) to 4.1 (± 11.9) events per hour (*p* = .002). Ten days of sick leave was enough for 81.3% of patients.

**Conclusion:**

In adults with large tonsils and OSA, removing 90% of tonsil tissue may be effective. After intracapsular surgery, ten days may be a sufficient recovery time.

**Supplementary Information:**

The online version contains supplementary material available at https://doi.org/10.1007/s00405-026-10497-1.

## Introduction

Obstructive sleep apnoea (OSA) causes cardiovascular morbidity, decreases in cognitive function, a lowered quality of life, and premature death [1]. Typical symptoms, such as loud snoring, excessive daytime sleepiness, and sleep-maintenance insomnia, prompt many patients to seek treatment [2]. In adults, the prevalence of OSA is rising [3]. Continuous positive airway pressure is the gold standard treatment, but 25% of patients are non-adherent to this therapy and seek other treatment options, including surgery [4, 5].

Among patients with large tonsils and especially with an apnoea-hypopnoea index (AHI) of less than 30 per hour, isolated traditional extracapsular tonsillectomy (ECTE) is an effective treatment option for adult OSA [6]. Sleep surgery studies most often use Sher’s criteria, where surgical success is defined by a greater than 50% decrease in the overall AHI and a decrease in the overall AHI to less than 20 [7]. In prior tonsillectomy studies on patients with large tonsils, surgical success rates range from 78 to 85.2% [6, 8]. In sleep surgery, a tonsillectomy is often combined with an uvulopalatopharyngoplasty, but in these select patients with tonsillar hypertrophy, additional soft palate surgery seems to have minimal added benefit [9–11].

In children suffering from sleep-disordered breathing or paediatric OSA, intracapsular tonsillectomy (ICTE) or tonsillotomy is commonly utilized. Multiple studies in the paediatric population show that it has equal efficacy, while postoperatively, children have less pain and fewer postoperative complications [12, 13].

In contrast, in adults, the effectiveness of tonsillotomy or ICTE remains unknown in the treatment of OSA. As already well-documented in children, in adults, ICTE also seems to be preferable to ECTE in terms of pain, complications, and patient satisfaction [14]. After tonsillotomy, in adults, one prior study suggests that a return to work could happen up to 7 days earlier than after tonsillectomy [15]. Prior studies of ICTE in the treatment of sleep-disordered breathing in adults focus solely on patients’ self-reported metrics [14]. Notably, a knowledge gap remains concerning postoperative AHI values.

This study is the first to report pre- and postoperative AHI values to more accurately assess the efficacy of ICTE in adults with OSA. This study was designed to be a pilot prospective case series to explore if conducting a randomised controlled trial comparing ECTE and ICTE should be considered. Additionally, we assessed if 10 days of sick leave after ICTE would suffice instead of the 14 days patients receive after ECTE.

## Materials and methods

The study protocol was reviewed and approved by the Medical Ethics Committee of the Hospital District of Southwest Finland (No. ETMK11/1801/2020), and it had institutional approval. All participants provided written informed consent.

### Participants and trial design

This observational study was a prospective case series with planned data collection. Adult patients with OSA presenting to recruiting centres (one secondary, one tertiary) were evaluated, and all eligible patients were invited to participate. All patients had home sleep apnoea test (HSAT) results available before evaluation for inclusion. Recruitment lasted from September 2020 to January 2025. Inclusion criteria included an AHI ≥ 15, hypertrophic tonsils (3–4/4 Brodsky), and an age between 16 and 65 years old. Exclusion criteria included body mass index (BMI) ≥ 35, untreated symptomatic nasal blockage, untreated symptomatic gastric reflux, prior pharyngeal surgery, and a diagnosis of malignancy or bleeding disorders. All patients were treated with ICTE. All HSAT were (re)interpreted, when possible, by a single certified scorer (MS). After this reinterpretation, two patients’ preoperative AHI were reduced to below 15, and they were included.

### Intervention

**Fig. 1:**
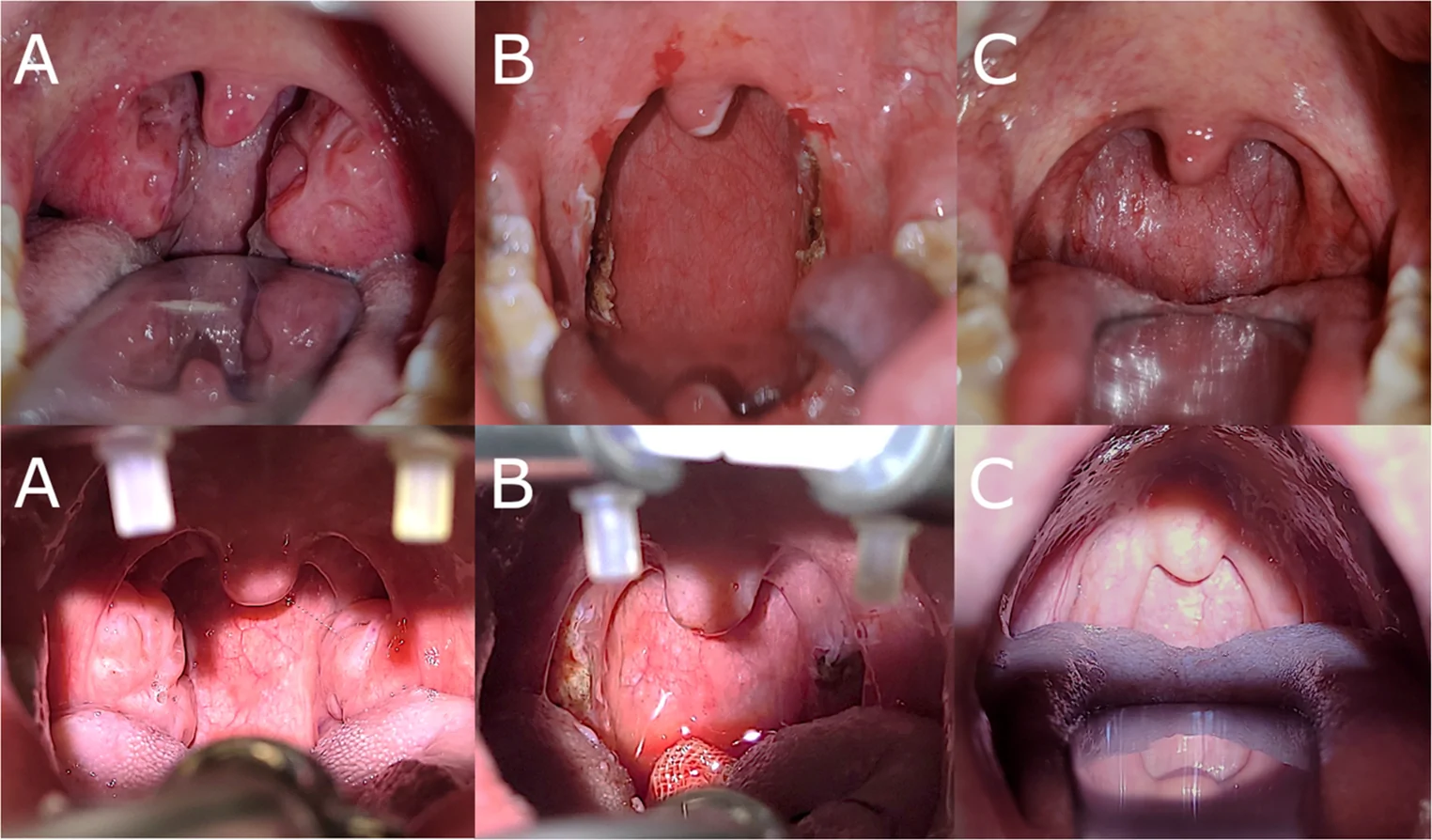
The preoperative, postoperative, and 6-months follow-up pictures of 2 patients after intracapsular tonsillectomy; **A **= preoperative; **B **= immediate postoperative; **C **= at 6-months follow-up; all intracapsular tonsillectomies were performed with bipolar temperature-controlled radiofrequency thermal ablation, i.e., coblation, to remove 90% of tonsil tissue

All surgeries were performed within 6 months of recruitment with bipolar temperature-controlled radiofrequency thermal ablation, i.e., coblation, aiming for 90% removal of tonsil tissue and leaving the tonsillar capsule untouched. All surgeries were performed by one of three surgeons (HS, JK, LLT). Patients were photographed preoperatively, immediately postoperatively, and at 6‑month follow-up. Figure 1 presents these images for two patients. At discharge, all patients were prescribed naproxen and a combination of tramadol-paracetamol for analgesia. Postoperative HSAT were performed 6 months after surgery, all with the same device (NoxT3).

### Questionnaires and surveys 

All participants completed pre- and post-operative quality-of-life questionnaires, including the Insomnia Severity Index (ISI), Epworth Sleepiness Scale (ESS), and the Glasgow Benefit Inventory (GBI) [16–18]. To track recovery, participants completed the Brief Pain Inventory (BPI) for 21 days postoperatively and the Nordic Tonsil Surgery Register (NTSR) 1-month questionnaire [19, 20]. At the 6-month follow-up, the patients also answered a 1-question survey of whether they would still choose the same surgery. Adverse events were recorded through electronic medical journal systems in addition to the NTSR 1-month questionnaire.

### Outcomes

As defined by Sher’s criteria as a > 50% decrease in the overall AHI and a decrease in the overall AHI to < 20 in HSAT, the primary outcome was surgical success. Surgical cure was defined by a decrease in the overall AHI to < 5. The patients were informed that routine sick leave after tonsillectomy is 14 days, were given 10 days of sick leave, and instructed that they would receive an additional 4 days, no-questions-asked, if needed, with a phone call. Additional HSAT data (i.e., snoring, AI, ODI3, SpO_2_), ESS scores, ISI scores, GBI scores, and the satisfaction rate were also evaluated. Pain interference levels and pain medication use were evaluated using BPI.

### Statistical analysis

Wilcoxon matched-pairs signed-rank tests were used to test each variable to assess for a change from pre- to postintervention. Patients were stratified into two sets of two groups both by surgical success and surgical cure statuses, and significant differences in age, BMI, and preoperative AHI-values between groups were assessed with the Mann-Whitney U test. Patients were then divided into two groups based on whether their preoperative AHI exceeded 30. Fisher’s exact test was used to assess the association of smoking status and preoperative AHI groups on surgical outcomes (i.e., surgical success and cure). A logistic regression was performed to ascertain the effects of age and weight on the likelihood of surgical cure. All statistical analyses were completed with IBM SPSS version 29.0.0 (IBM Corp, New York).

## Results

Twenty patients with moderate to severe OSA completed enrolment and intervention from October 2020 to January 2025. Sixteen patients (4 female, 12 male) completed the 6-month follow-up with HSAT and were included for further analysis. The mean age was 31.4 years (range 17.8–47.5). On the day of surgery, tonsil grades were IV in 4 patients, III in 11, and II in 1 (grade III at recruitment). Mean BMI was 29.4 (SD ± 4.5) and mean 6‑month weight change was + 1%. No patient lost ≥ 10% body weight (exclusion criterion).

**Fig. 2:**
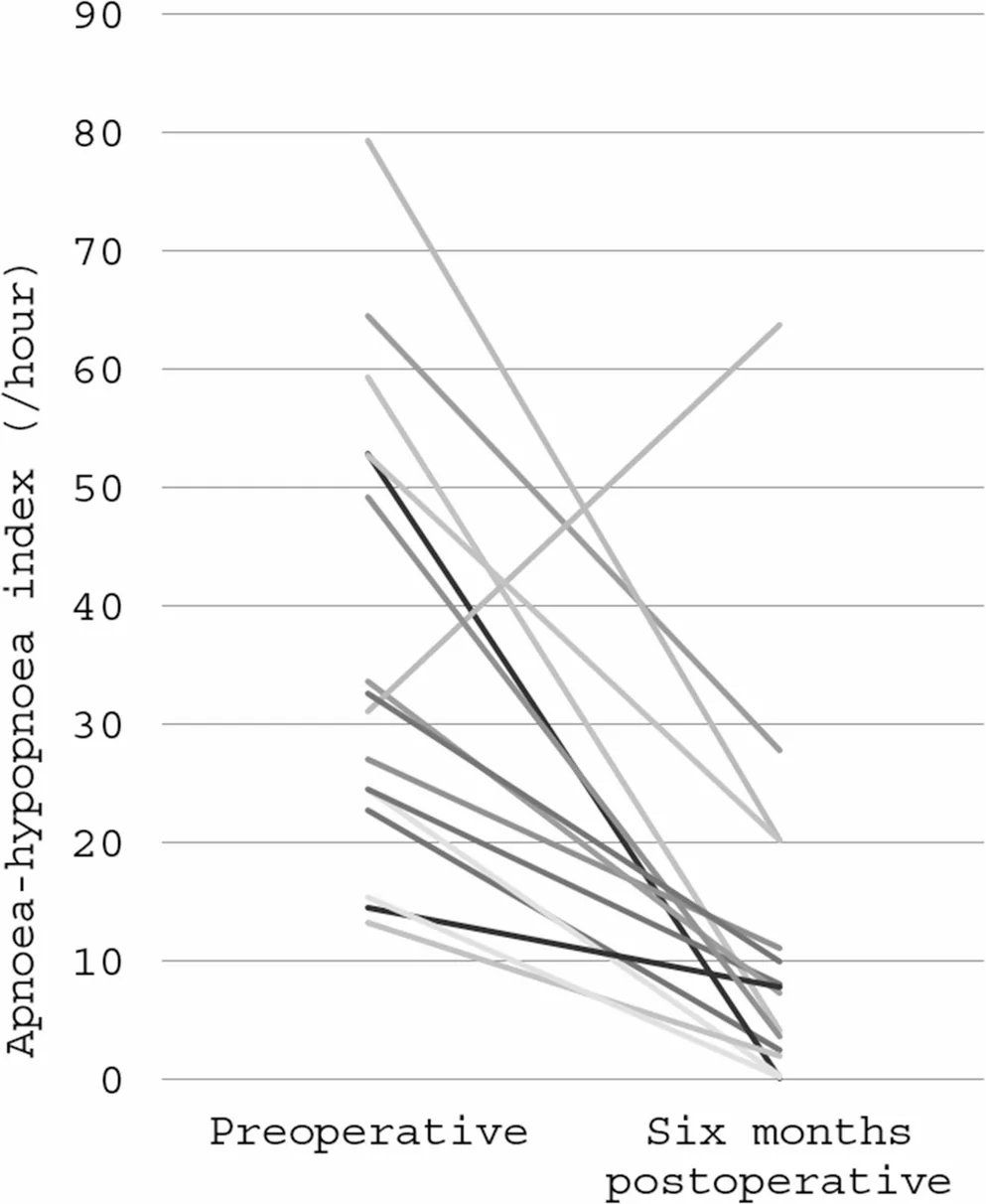
Individual apnoea-hypopnoea indexes (reported number is events per hour) before and 6 months after intracapsular tonsillectomy, and the line graphs represent the individual values

The surgical success rate was 69% (95% CI 43–94). The surgical cure rate was 44% (95% CI 16–71). For 13 of 16 patients (81%), ten days of sick leave was sufficient for recovery. The mean (± SD) AHI decreased from 37.4 (± 19.8) to 11.8 (± 16.1) events/h (*p* = .003, Cohen’s d = 1.5), corresponding to an average reduction of -25.5 (± 22.5) events/h or 67% (95% CI 41–93). In Fig. 2; Table 1, pre- and postoperative AHI of each patient is shown. The electronic data supplement contains all pre- and postoperative HSAT data gathered.

**Table 1:** Pre- and postoperative home sleep apnoea test data

ID		Pre-operative			At 6 months			AHI change			
	Tonsil grade	AHI (/h)	Supine AHI (/h)	AI (/h)	AHI (/h)	Supine AHI (/h)	AI (/h)	Per hour	Per-cent	Success^a^	Cure
4	3	22.8	27.5	5.9	2.5	4	0	-20.3	-89.0	Yes	Yes
7	4	59.3	N/A	6	4.1	1.8	0.1	-55.2	-93.1	Yes	Yes
10	3	64.5	N/A	50	27.8	113.1	4.8	-36.7	-56.9	No	No
11	4	52.9	57.9	24.9	0.1	0.4	0	-52.8	-99.8	Yes	Yes
12	3	24.5	56.9	7.9	0.4	1.7	0	-24.1	-98.4	Yes	Yes
13	3	79.4	74.2	N/A	20.2	29	4.6	-59.2	-74.6	No	No
14	2	27.1	39.2	1.7	11.1	17	0.4	-16	-59.0	Yes	No
16	3	24.6	53.3	8.3	8.1	34.2	1.4	-16.5	-67.1	Yes	No
18	3	13.3	40.3	4	2	0.8	0	-11.3	-85.0	Yes	Yes
19	4	33.7	70.2	N/A	7.3	7.5	0.6	-26.4	-78.3	Yes	No
20	3	14.5	19.3	0	7.8	9.6	0	-6.7	-46.2	No	No
21	4	15.4	18.2	6.3	0.2	0.5	0	-15.2	-98.7	Yes	Yes
22	3	31.1	47.4	N/A	63.8	59.7	48.2	+ 32.7	+ 105.1	No	No
23	3	49.2	86.1	11.5	3.6	1.8	0	-45.6	-92.7	Yes	Yes
24	3	32.7	35.5	19.3	10	22.1	0.5	-22.7	-69.4	Yes	No
25	3	52.8	107.7	27.1	20.2	100.6	5	-32.6	-61.7	No	No
Mean	3.2	37.4	52.4	13.3	11.8	25.2	4.1	-26.0	-66.9		
Std. deviation	± 0.53	± 19.8	± 25.7	± 13.9	± 16.1	±35.8	± 11.9	± 22.5	± 48.7		

^a^>50% decrease in the overall *AHI* and a decrease in the overall *AHI* to < 20

*AHI* Apnoea-hypopnoea index. events per hour

0–6 months difference p-values, *AHI* 0.003. Supine *AHI* 0.002. *AI* 0.002

While supine, the mean AHI lowered from 52.4 (± 25.7) to 25.2 (± 35.8) events/h (*p* = .002). The mean AI lowered from 13.3 (± 13.9) to 4.1 (± 11.9) events/h (*p* = .002). The mean ODI3 lowered from 36.9 (± 20.3) to 11.3 (± 14.4) events/h (*p* = .001). The mean minimum SpO₂ increased from 82.4% (± 6.7) to 87.1% (± 4.1, *p* = .008). The average time spent under 90% SpO₂ decreased from 5.9 (± 7.0) to 2.4 (± 5.6) minutes (*p* = .04). The mean changes in total snoring, supine snoring, and average SpO₂ were not significant.

**Fig. 3:**
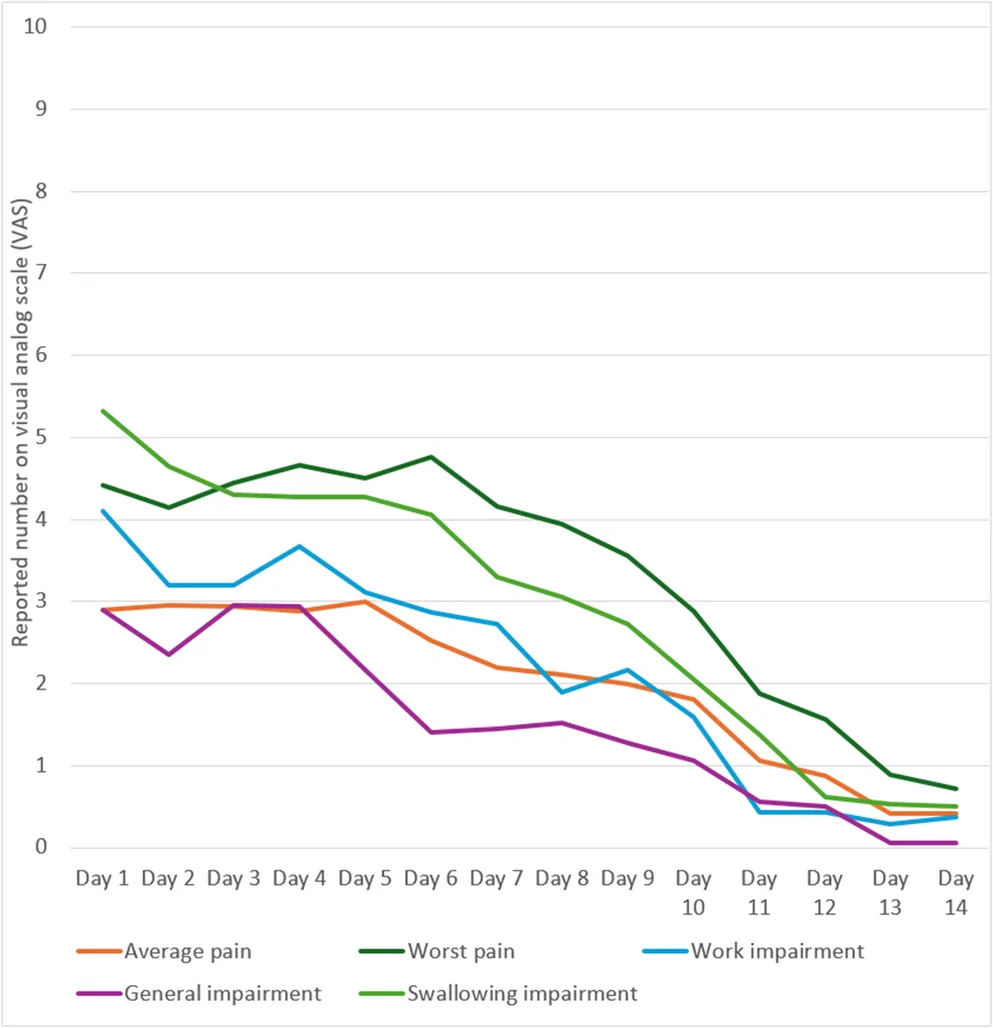
Average postoperative VAS scores from the pain questionnaire (days 1–14, patients *N* = 16) During the last 24 h, on a scale from 0 to 10, how much… Average pain = was the pain on average? Worst pain = was the pain at its worst? Work impairment = did the pain affect your ability to work? General impairment = did the pain affect your general performance? Swallowing impairment = did the pain affect your swallowing?

In BPI, patients reported that on postoperative day 10, the most intense pain during the day was a mean (± SD) of VAS 2.6 (± 1.9) out of 10, and the average pain throughout the same day was 1.7 (± 1.5) out of 10. On day 6, the highest mean (± SD) intake of tramadol-paracetamol-combination tablets per patient was 3.2 (± 2.2) tablets out of maximum 8. By day 10, the mean intake was 1.3 (± 2.0) tablets. The daily average pain VAS never rose above 3.0 (± 1.9) during recovery. The daily average pain VAS scores and their effect on patients’ ability to work, general performance, and swallowing are reported in Table 2; Fig. 3. In Table 2, the mean daily naproxen and tramadol-paracetamol tablet intake is also reported.

**Table 2:** Pain interference on a visual analogue scale 0–10 (mean [± SD]) and usage of pain medication (mean [± SD]) daily for 14 days after intracapsular tonsillectomy, from Brief Pain Inventory data

During the last 24 h, on a scale from 0 to 10, how much:	Day 1	Day 2	Day 3	Day 4	Day 5	Day 6	Day 7	Day 8	Day 9	Day 10	Day 11	Day 12	Day 13	Day 14
was the pain on average?	2.9 (± 1.3)	3.0 (± 1.5)	2.9 (± 1.4)	2.9 (± 1.4)	3.0 (± 1.9)	2.5 (± 1.5)	2.2 (± 1.4)	2.1 (± 1.7)	2.0 (± 1.5)	1.8 (± 1.4)	1.1 (± 0.9)	0.9 (± 0.9)	0.4 (± 0.7)	0.4 (± 0.7)
was the pain at its worst?	4.4 (± 2.1)	4.2 (± 2.0)	4.5 (± 1.9)	4.7 (± 1.9)	4.5 (± 2.0)	4.8 (± 2.2)	4.2 (± 2.2)	3.9 (± 2.3)	3.6 (± 2.2)	2.9 (± 1.9)	1.9 (± 1.4)	1.6 (± 1.5)	0.9 (± 1.2)	0.7 (± 1.1)
did the pain affect your ability to work?	4.1 (± 3.5)	3.2 (± 3.1)	3.2 (± 3.2)	3.7 (± 3.3)	3.1 (± 2.7)	2.9 (± 2.8)	2.7 (± 3.1)	1.9 (± 2.9)	2.2 (± 3.0)	1.6 (± 2.2)	0.4 (± 1.0)	0.4 (± 1.1)	0.3 (± 1.0)	0.4 (± 1.0)
did the pain affect your general performance?	2.9 (± 2.4)	2.4 (± 2.2)	3.0 (± 2.0)	2.9 (± 2.0)	2.2 (± 1.8)	1.4 (± 1.5)	1.4 (± 1.5)	1.5 (± 1.8)	1.3 (± 1.8)	1.1 (± 2.0)	0.6 (± 1.7)	0.5 (± 1.3)	0.1 (± 0.2)	0.1 (± 0.2)
did the pain affect your swallowing?	5.3 (± 2.4)	4.7 (± 2.2)	4.3 (± 1.9)	4.3 (± 2.0)	4.3 (± 2.1)	4.1 (± 2.0)	3.3 (± 2.0)	3.1 (± 2.3)	2.7 (± 2.0)	2.1 (± 1.6)	1.4 (± 1.4)	0.6 (± 1.1)	0.5 (± 1.0)	0.5 (± 0.9)
*How many tablets have you taken during the last 24 h of*:														
Naproxen 500mg^a^	1.1 (± 0.7)	1.6 (± 0.7)	1.6 (± 0.7)	1.6 (± 0.7)	1.6 (± 0.7)	1.7 (± 0.6)	1.6 (± 0.8)	1.4 (± 0.8)	1.5 (± 1.1)	1.3 (± 0.9)	0.8 (± 0.9)	0.7 (± 0.9)	0.6 (± 0.9)	0.5 (± 0.8)
Tramadol 37.5 mg / Paracetamol 325mg^b^	1.9 (± 1.6)	2.9 (± 1.9)	3.1 (± 2.2)	3.1 (± 2.3)	2.9 (± 1.9)	3.2 (± 2.2)	2.8 (± 2.0)	2.3 (± 2.0)	1.7 (± 2.1)	1.3 (± 2.0)	0.8 (± 2.0)	0.6 (± 1.3)	0.4 (± 1.1)	0.5 (± 1.1)

Out of daily maximum allowance: ^a^ 2 tablets, ^b^ 8 tablets

Patients were instructed to primarily take naproxen and then add the tramadol/paracetamol combination tablet as needed

One patient self-reported seeing blood-tinged sputum at home during recovery but the bleeding was self-contained. No other adverse effects were recorded.

ESS did not change significantly with the mean (± SD) preoperative score being 6.1 (± 3.2) and the mean postoperative being 5.3 (± 2.8) (*p* = .17). ISI score lowered from a mean (± SD) preoperative score of 9.6 (± 3.4) to a mean postoperative score of 6.3 (± 4.0) (*p* = .005).

GBI responses were scaled and averaged to give a score with a range of − 100 (poorest outcome) to + 100 (best outcome). GBI questions were divided into three subscales (i.e., general, social, physical). The mean (± SD) scores were + 11.5 (± 10.2) for total, + 16.4 (± 11.9) for the general subscale, + 1.0 (± 9.3) for social support, and + 0.5 (± 31.0) for physical health. At 6 months, 100% of patients answered that they would still choose this surgical intervention.

In a secondary analysis, patients were stratified into two sets of two groups both by surgical success and surgical cure statuses. Because of the small sample size and because these subgroup analyses are exploratory the results should be approached with caution. Significant differences in age, BMI, smoking status, and preoperative AHI-values between groups were assessed. Significant differences were found in the surgical cure group in mean BMI (26.8 vs. 31.4, *p*=.050), mean age (24.5 vs. 36.8, *p*=.01), and smoking status (0% vs. 56%, *p*=.03) compared to no surgical cure groups. No significant differences were found in the mean preoperative AHI-values between surgical cure groups. No significant differences were found in the mean age, BMI, smoking status, and preoperative AHI-values between any surgical success compared to no surgical success groups.

When splitting the patients into two groups based on whether their preoperative AHI exceeded 30/h, no statistically significant difference in surgical success or surgical cure between these two groups was found. A binary logistic regression was conducted to examine the effect of age and BMI on the likelihood of surgical success. In a univariate logistic regression, age had a statistically significant effect on the outcome (*p* = .03, OR = 0.80 [95% C.I. 0.65–0.98]) suggesting that with increasing age, the odds decreased. BMI alone was not a significant predictor (*p* = .06). Age and BMI were moderately correlated (Spearman’s rank correlation rₛ = 0.40), and when both variables were included in the multivariable model, neither remained statistically significant (*p* = .051 [age], *p* = .16 [BMI]).

## Discussion

In adults with large (grade 3–4) tonsils, intracapsular tonsillectomy may be an effective treatment for obstructive sleep apnoea. The primary outcome was surgical success according to Sher’s criteria, which was achieved in 69% of patients. In prior tonsillectomy studies, surgical success rates range from 78 to 85% [6, 8]. Our success rate can also be compared to reported success rates of 52% for uvulopalatopharyngoplasty and 66–87% for barbed suture pharyngoplasty, while noting that these studies also included patients with smaller tonsils [21, 22].

Although our success rates fall slightly below prior studies of ECTE, the average reduction in AHI of -25.5 events per hour or -67% is comparable. In these, the mean reduction in AHI is reported as -30.2 events per hour or -65% [6]. For AHI, the minimal clinically important difference is generally considered to be a reduction of 15 events per hour, which, in this study, was reached in 13 patients (81%) [23].

Our results also suggest that, for adults, after ICTE, 10 days of sick leave from work is sufficient. As mentioned, one prior study on adults suggests returning to work after ICTE could happen up to 7 days earlier than after extracapsular tonsillectomy [15]. In this study, the mean pain interference on work, general performance, and swallowing remains modest during recovery, and the intake of mild opioids was under maximum allowance. A prior study also suggests that adults could recover from ICTE without any opioids [15].

Sleep apnoea patients often suffer from comorbid insomnia, and we saw a significant reduction in the ISI scores of patients after surgery possibly reflecting reduced waking arousals and fragmentation of sleep [24]. The postoperative mean score for this cohort fell within the range considered normal for individuals without insomnia, which is 0–7 [25]. Patients with lower sleepiness, such as those in our study, are more vulnerable to develop co-morbid insomnia [26]. The co-occurrence of insomnia and OSA is linked to greater impairments in both subjective and objective sleep quality and quantity, heightened daytime dysfunction, and reduced productivity and quality of life [24].

Prior tonsillectomy studies show significant effects on ESS [6, 8]. Our postoperative ESS were comparable, but the preoperative average score of 6.1 is already within the usual range seen in normal controls (5.9 ± 2.2), which explains the non-significant reduction [16]. In the literature, the correlation between ESS and AHI remains controversial and may be a discrepancy that may be pronounced in younger cohorts with moderate AHI scores [27]. ESS primarily measures hypersomnolence and correlates poorly with disease severity or in having a treatment benefit in certain patient groups. We have reported similar findings in tonsillectomy cohorts, and CPAP treatment reduces ESS by an average of only 1.2 points in patients with mild to moderate OSA [28, 29].

In this cohort, the mean GBI scores showed modest improvements in quality-of-life indexes. One prior study in snoring patients reported distinctly higher GBI outcomes, with a mean total score of 58 and a mean general subscale score of 40 [30]. Despite limited changes in GBI-derived quality-of-life measures, 100% of participants stated that they would elect to undergo ICTE again. Traditional Finnish cultural behavioural norms emphasize modesty and may affect how participants respond to self-reporting instruments.

Because of the small sample size, the subgroup analyses should be approached with caution. Significant differences were found between the surgical cure compared to the no surgical cure groups in the mean BMI, mean age, and in smoking status. The no surgical cure group had significantly older patients, patients with a higher BMI, and more smokers. We did not find similar differences between surgical success compared to no surgical success groups. In the treatment of OSA, previous studies show that increasing BMI and age are associated with reduced efficacy of tonsillectomy [6, 8]. Previous studies indicate that tonsillectomy is most effective in patients with preoperative AHI under 30/h, but we had no such findings [6]. In univariate logistic regression, the small cohort size limited statistical power, with only age reaching significance; a higher age lowered the effect of surgery. Nevertheless, our results suggest that BMI, age, and smoking status may serve as potential determinants of the likelihood of treating OSA with ICTE, and that aligns with results in previous sleep-surgery studies.

Given the sample size, two cases disproportionately affected our overall success rate (69%) despite favourable outcomes. Patient 13 showed a 75% AHI reduction (-59.2/h) but had a postoperative AHI of 20.2/h. Patient 20 had a postoperative AHI of 7.8/h yet only had a 46% reduction. Of interest, patient 14, whose tonsils were grade II on the day of surgery, was a surgical success. The only patient whose sleep apnoea worsened during the study, patient 22, had severe sleep apnoea with challenging HSAT results to quantify because of probable wake periods and consecutive hypopneas and apnoeas with poorly differentiated desaturations. Changes in these features between pre-operative and different post-operative HSAT are the most likely reasons for the unusually variable AHI indexes.

A limitation of the surgical technique employed in this study is the inability to obtain objective volumetric measurements of the resected tissue. Whether objective tonsil volume or subjective tonsil grade is the stronger predictor of surgical success remains a matter of debate in the literature [31, 32].

Despite being a prospective cohort, this study has several limitations. To establish noninferiority to tonsillectomy, a randomised controlled trial would have been required. Due to the limited number of eligible patients at recruiting centres, this was impractical. Preoperative HSAT was not repeated if a recent study was available, resulting in some variability in recording equipment across patients. Also, our cohort size was small. As with all nonrandomized studies, the potential for selection bias exists. The uncontrolled study design increases the risk of confounding variables influencing outcomes. Selection bias occurred among patients referred to the participating centres. Despite multiple follow-up attempts, four of the twenty enrolled patients did not complete the 6-month at-home sleep apnoea test. Additionally, we did not assess the Friedman Tongue Position or Mallampati grade preoperatively, although they are known to influence outcomes in sleep surgery [33]. Drug-induced sleep endoscopy was also not performed, as it was not conducted at our institution at the time of this trial. Additionally, HSAT are known to have limitations in event detection accuracy, may underestimate or misclassify respiratory events, and night-to-night variability is large [34]. Long-term outcomes are not yet available, but this cohort will be followed for up to five years.

## Conclusion

This study suggests that ICTE may be effective in treating adult OSA in patients with large tonsils. The HSAT results are promising and warrant a prospective randomized clinical trial between tonsillectomy and ICTE to explore noninferiority in adults. 10 days sick leave after ICTE may be adequate for most patients.

## Supplementary information

Below is the link to the electronic supplementary material.

Supplementary Material 1
